# Novel Magnetic Properties in Curved Geometries

**DOI:** 10.3390/nano12071175

**Published:** 2022-04-01

**Authors:** Cristina Bran

**Affiliations:** Instituto de Ciencia de Materiales de Madrid (ICMM-CSIC), 28049 Madrid, Spain; cristina.bran@icmm.csic.es

The expanding of planar magnetic structures into three dimensions (3D) creates the possibility of tuning the conventional magnetic textures or producing novel effects and functionalities by tailoring their curvature [1]. The effect of curvature in the magnetic systems is an emerging topic of research because of the novel magnetization textures and dynamics related to topology as compared to planar geometries. The interesting capabilities of 3D magnetic structures with curved geometry come in the first place from the enormous increase in the active surface for storage, as well as from their versatile geometrical forms, resulting in complex 3D magnetic configurations with great potential for manipulation [2]. 

One of the most promising magnetic structures with curved geometry are cylindrical magnetic wires, as they offer multifunctional responses to electric/ magnetic fields, electric current, mechanical stress, or thermal gradients and thus can be used for the interconversion between different functionalities.

Their circular symmetry determines multiple topologically non-trivial magnetization structures, such as Bloch-point domain walls, which are faster and more stable than domain walls in planar structures, or helical magnetic configurations, vortices, and skyrmion tubes [3,4].

Exploiting these unusual three-dimensional magnetic configurations and their dynamics can lead to advanced applications such as new-generation spintronic-based magnetic recording, bio-magnetics, robotics, sensors, and actuators devices [5].

This Special Issue of *Nanomaterials* covers the recent advancements in the fabrication, characterization, and potential technological applications of magnetic wires, as single magnetic structures or as part of 3D ordered architectures, in magnetic sensors and data storage, microwave devices, or thermomagneto-electric devices.

The first review [6] of the Special Issue presents many examples of how the magnetic structure can be tailored and controlled in cylindrical nanowires by geometry and magnetocrystalline anisotropy or manipulated by magnetic fields and spin-polarized currents. The nanowires are prepared by electrochemical methods into the porous pores of alumina templates, allowing the fabrication of magnetic nanowires with precise control over geometry, morphology, and composition. The diameter modulations change the typical single domain state present in crystallographic cubic nanowires, providing the possibility to confine or pin circular domains or domain walls in each segment. The control and stabilization of domains and domain walls in cylindrical wires have been achieved in multi-segmented structures by alternating magnetic segments of different magnetic properties (producing alternative anisotropy) or with non-magnetic layers. The results point out the relevance of the geometry and magnetocrystalline anisotropy to promote the occurrence of stable magneto-chiral structures and provide further information for the design of cylindrical nanowires for multiple applications [6].

Similarly, by playing with the architecture of cylindrical magnetic nanostructures, J. Garcia el al. [7] presents a magnetic study on core/shell nanostructures formed by a nanowire nucleus (Fe_56_Co_44_), grown by electrodeposition and coated by a non-magnetic SiO_2_ layer coaxially surrounded by a magnetic Fe_3_O_4_ nanotubular coating both fabricated by means of the Atomic Layer Deposition technique. The magnetic reversal studied by First Order Reversal Curve methodology reveals a two-step magnetization reversal of the core/shell bi-magnetic nanostructure. These results are also confirmed by the hysteresis loops of individual core/shell nanostructures measured by Kerr effect-based magnetometer.

Gomes et al. [8], present an interconnected 3D nanowire system fabricated by direct electrodeposition in track-etched polymer templates with crossed nano-channels. This method allows the fabrication of crossed single element and multilayered nanowires with controlled morphology and material composition. The interconnected nanowire networks exhibit high room-temperature Seebeck coefficient and power factors, making them promising candidates for the next generation of flexible and lightweight thermoelectric devices [8].

An alternative method to produce curved 3D nanostructures with defined and complex geometries is presented in the review paper by C. Magen et al. [9]. Focused electron beam induced deposition (FEBID) is a single-step additive nanolithography technique based on the local decomposition of the molecules of an organometallic precursor gas, adsorbed on the surface of a substrate, thus producing a solid deposit. The review presents different possibilities for engineering the geometrical, compositional, and magnetic properties of 3D ferromagnetic nanowires grown by FEBID, focusing on the fine-tuning of FEBID growth parameters to modify the composition and dimensions, the growth of core-shell heterostructures, and the purification by thermal annealing.

An important part of this Special Issue deals with the magnetic microwires. The peculiarities of microwires circular symmetry, outstanding magnetic properties, and the cheap and accessible fabrication method makes them attractive from sensing applications.

The review article by Chizhik et al. [10] summarizes the results of the magnetic, magneto-electric, and magneto-optical studies of a series of glass-covered microwires. The experimental data are complemented by micromagnetic simulations. Different tools for controlling the reversible and irreversible transformations of the magnetic structure of glass-covered microwires are evidenced. The irreversible tools are the selection of the chemical composition, geometric ratio, and the stress-annealing, while a combination of magnetic fields and mechanical stresses is needed for the reversible tuning. The study is also focused on the giant magnetoimpedance effect and the velocity of the domain walls propagation important for the technological applications.

Alekhina et al. [11] propose an indirect method for the estimation of micromagnetic structure in glass-coated microwires. The cross-sectional permeability distribution in the microwires was obtained from impedance measurements at different frequencies. This distribution enables estimation of the prevailing anisotropy in the local region of the wire cross-section. The results obtained were compared with the findings of magnetostatic measurements and remanent state analysis. The advantages and limitations of the methods are also discussed.

The Magnetoimpedance (MI) of Co-based microwires with an amorphous and partially crystalline state are investigated at elevated frequencies by Alam et al. [12]. Two mechanisms of MI sensitivity related to the DC magnetization re-orientation and AC permeability dispersion are discussed. Remarkable sensitivity of impedance changes with respect to applied tensile stress at GHz frequencies are obtained in partially crystalline wires subjected to current annealing. Increasing the annealing current enhanced the axial easy anisotropy of a magnetoelastic origin, which made it possible to increase the frequency of large-stress MI.

This collection of interesting research papers shows some of the capabilities and challenges in the preparation, characterization, and possible applications of magnetic wires with cylindrical geometry. 

Both from an experimental and micromagnetic modelling point of view, this Special Issue present interesting ideas, showing the prospects and exciting developments in the field of cylindrical magnetic wires.

Finally, I would like to express my gratitude to all the authors for their valuable contribution to this Special Issue.

## Data Availability

Not applicable.
